# Clinical Significance of CENP-H Expression in Uterine Cervical Cancer

**DOI:** 10.7497/j.issn.2095-3941.2012.03.007

**Published:** 2012-09

**Authors:** Mei-ying Weng, Lin Li, Shun-jia Hong, Shu-ying Feng

**Affiliations:** Department of Obstetrics and Gynecology, Sun Yat-sen Memorial Hospital of Sun Yat-sen University, Guangzhou 510120, China

**Keywords:** uterine cervical cancer, uterine cervix, CENP-H, immunohistochemistry, tumor staging, prognosis

## Abstract

**Objective:**

This work aims to investigate the expression pattern and clinicopathologic significance of centromere protein H (CENP-H) in uterine cervical cancer (UCC).

**Methods:**

The level of CENP-H expression in the paraffin sections of 62 UCC cases was determined by the SP immunohistochemical method, with complete clinicopathologic data in all cases. Statistical analysis was conducted to evaluate the prognostic and diagnostic significance of CENP-H using SPSS13.0 software package.

**Results:**

Immunohistochemical assay showed strong CENP-H expression in 61.29% (38/62) of the paraffin-embedded cervical cancer tissues. Statistical analysis revealed a strong correlation between the CENP-H expression and the clinical classification (*P*=0.038) of the cervical carcinoma. The expression increased with rise of the stages. The analysis of Cox proportional hazards regression model suggested that CENP-H expression (*P*=0.002) and tumor stage (*P*=0.001) were independent prognostic markers for the survival of UCC patients. The survival analysis showed that the survival rate was significantly lower in patients with high expression of CENP-H than in those with low expression of CENP-H (*P*=0.001).

**Conclusions:**

CENP-H is likely to be a valuable marker for carcinogenesis and progression of UCC. It might be used as the important diagnostic and prognostic marker for cervical carcinoma patients, especially for those at early stage.

## Introduction

The uterine cervical cancer (UCC) is a malignant tumor of top incidence in gynecological oncology. Moreover, there was a tendency of increase in its incidence over the past few years^[^^1^^]^. Nowadays, the combined therapy with surgery and radiation and chemotherapy fails to significantly raise the 5-year survival rate of UCC patients. Therefore, to investigate the sensitive and specific tumor marker, especially the early tumor marker, has an important role in the clinical diagnosis and treatment of tumors. The centromere protein H (CENP-H) is a structural protein in the inner plate of centromere, which sustain a correct segregation of the sister chromatid into the daughter cell during a process from the mitosis metaphase to mitosis anaphase of the cells^[^^2^^]^.

The low expression of CENP-H results in the cell division arrest at the metaphase of the mitosis. The high expression of the protein may bring about occurrence of the chromosome aneuploid in the disrupt daughter cells^[^^3^^]^. In the past few years, it has been found by some authors that several malignant tumors presented high CENP-H expression^[^^4^^-^^6^^]^. At the same time, others discovered that there was a correlation between the expression of CENP-H and prognosis of patients with nasopharyngeal carcinoma (NPC)^[^^6^^]^. In our study, immunohistochemical staining method was used to investigate the expression of CENP-H in UCC, and the relationship between the level of CENP-H expression and the clinicopathologic features. The correlation of expression of CENP-H with the oncogenesis and progression of UCC, as well as the relationship between CENP-H and the prognosis of UCC patients were explored in this study.

## Materials and Methods

### Materials

The clinicopathologic data of 62 UCC cases were selected from the paraffin-embedded histological specimens which were obtained by surgery and pathologically proven in the No. 2 Hospital of Sun Yat-sen University, also named as Sun Yat-sen Memorial Hospital of the University, Guangzhou, during a period between January 2006 and January 2010. The follow-up data were complete in all cases, with a follow-up rate of 100%. The shortest follow-up period was 8 months, and the longest was 60 months, with 26 on average. The age of patients at first visit ranged from 32 to 82 years, with a mean age of 54. By the end of the follow-up (December 2010), 29 of the 62 patients died, among which 21 died of tumor recurrence and 8 died of distant metastasis of the tumor. All the other 33 patients alive were with disease-free survival. Based on the up-to-date 2002 TNM classifications of International Union Against Cancer (UICC)^[^^7^^]^, 23 of the total cases were with stage T_1_/T_2_, and 39 were with stage T_3_/T_4_. Lymph node metastasis was not found in 35 of the cases, but was seen in 27 others. Of the total cases, 26 were with stage-I/II and the other 36 with stage-Ш/IV.

### Reagents

The rabbit-antihuman CENP-H monoclonal antibodies were bought from the Bethyl Lab, USA, the SP immunohistochemical kits were bought from the Beijing Zhongshan Golden Bridge Biotechnology Co. (BZGBBC) and the antibody diluents were also purchased from the BZGBBC.

### Methods

A 4-µm paraffin section was used for serial sections in the immunohistochemical stain. First, the tissue slice was put in the xylene for deparaffinage, 10 min each time and 2 times in all. Ethanol gradient elution was used for hydration, 3% H_2_O_2_ was used for eliminating the endogenous peroxidase. EDTA (pH 8.0) antigen reparative liquid was used for routine repair: the section was put in a microwave oven with high heat for 5 min, thawed out for 2 min and heated (medium-low) for 20 min, so as to sufficiently expose the antigen site. After self-cooling, liquid A (glacial acetic acid plus sulfuric acid) was added in the oven, with blocking of the section at room temperature for 15 min. The rabbit-antihuman CENP-H (1:100, Bethyl, USA) was incubated overnight at 4°C, with 1×PBST (1‰Tween) rinsing for 3 times, 5 min each time. Then, the liquid B (peracetic acid) (SP KIT from BZGBBC) was added, and was incubated for 15 min at room temperature, with 1×PBST (1‰Tween) rinsing for 3 times, 5 min each. Next, the liquid C (SP KIT from BZGBBC) was added and was incubated at room temperature for 15 min, with 1×PBST (1‰Tween) rinsing 3 times, 5 min each. DAB staining was conducted for 10 min. Following Hematoxylin afterstain and ethanol/alcohol hydrochloride fixation, the section was observed. The antibody diluent was used to replace the first antibody as the negative control. The glass for positive control was provided by Bethyl Lab, USA.

The level of staining in the histological specimen was observed by optical microscope. The expression of CENP-H protein presented as brownish-yellow or dark brown granules was seen in the cell nucleus. The scoring criterion reported by Liao et al.^[^^6^^]^ was used. The total cell count and nucleus-positive cell count ranged from 0 to 100% based on the percentage of the positive cells. Meanwhile, the scoring was done based on the degree of the staining intensity, i.e., the score of 0 was for no staining, 1 for slight staining (faint yellow color), 2 for moderate staining (brownish yellow), and 3 for strong staining (brown). A staining of ≥2 scores and above, and with positive cells of ≥50% was regarded as high expression of CENP-H. The positive cells of <50% or the score of staining of <2 scores indicated low expression of CENP-H. The above results were confirmed by two pathologists using the double blind method.

### Statistical analysis

The SPSS 13.0 software package was used for analysis of the data. The χ^2^ test was used to analyze the relationship between CENP-H expression and clinicopathologic factors. Multivariable cox regression analysis was conducted for screening the risk factors affecting the survival of UCC patients. Log rank test and Kaplan-Meier analysis were utilized for analyzing the survival rate. *P*<0.05 indicates significant differences.

## Results

### Expression of CENP-H in UCC

**Figure 1:**
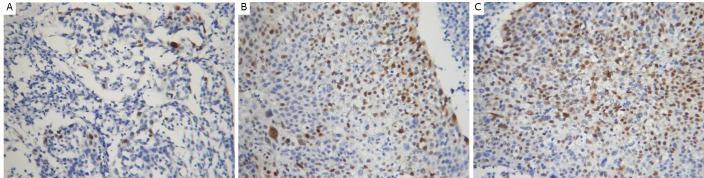
Immunohistochemical staining for CENP-H expression in uterine cervical cancer and paraneoplastic tissues. A: Absence of CENP-H expression in paraneoplastic tissues; B: Low expression of CENP-H in uterine cervical cancer; C: High expression of CENP-H in uterine cervical cancer (200 ×).

CENP-H expressed in the nucleus of UCC cells. However, CENP-H staining of the cell nucleus was not found in the paraneoplastic epithelial cells (**Figure 1**). In the 62 UCC specimens, the rate of high CENP-H expression was 61.29% (38/62).

### Relationship between expression of CENP-H and clinicopathologic features of UCC

There was no statistical significance in the difference of CENP-H expression among various age groups (*P*=0.906). There was statistical significance in the difference of CENP-H expression among various clinical stages of UCC (*P*=0.038). There was a positive correlation between the clinical staging of tumor and high expression of CENP-H (*r*=0.264, *P*=0.038). Expression of CENP-H was highly correlated with tumor size (*P*<0.05). There were no statistically significant difference in the high expression of CENP-H among UCC of various T-stages (*P*=0.258). There was no significant difference in CENP-H expression between the group with nodal metastasis and that without nodal metastasis (*P*=0.773) (**Table 1**).

**Table 1:** Relationship between CENP-H expression and clinicopathologic features of UCC.

Characteristics	Cases	CENP-H		*P*
		Low expression (%)	High expression (%)	
Age (years)				
<60	29	11 (37.9)	18 (62.1)	0.906
≥60	33	13 (39.4)	20 (60.6)	
Tumor size (cm)				
≥4	24	6 (25.0)	18 (75.0)	0.001
<4	38	18 (47.4)	20 (52.6)	
Stage				
I/II	26	14 (53.8)	12 (46.2)	0.038
III/IV	36	10 (27.8)	26 (72.2)	
T-staging				
T_1_/T_2_	23	11 (47.8)	12 (52.2)	0.258
T_3_/T_4_	39	13 (33.3)	26 (66.7)	
Nodal metastasis				
Existent	27	11 (40.7)	16 (59.3)	0.773
Nonexistent	35	13 (37.1)	22 (62.9)	

### Survival analysis

Cox regression analysis showed that the clinical staging of UCC was the independent prognostic factor (*P*=0.001). The coefficient of relative risk (RR) was 2.135. Also, the level of CENP-H expression was the independent prognostic factor of UCC (*P*=0.002), RR=1.490. CENP-H expression, T classification and lymph node metastasis were recognized as independent prognostic indicators for survival (**Table 2**).

**Table 2:** Univariate and multivariate cox regression analysis of the prognostic factors of UCC patients.

		Univariate analysis			Multivariable analysis		
	Cases	*P*	Regression coefficient (SE)		*P*	Coefficient of relative risk	95% CI
Stage							
I/II	26	0.003	1.104 (0.376%)		0.001	2.135	1.271-3.586
III/IV	36						
CENP-H							
High expression	38	0.001	0.917 (0.358%)		0.002	1.490	1.113–1.995
Low expression	24						
T-staging							
T_1_/T_2_	23	0.004	0.697 (0.114%)		0.007	1.280	0.971-1.428
T_3_/T_4_	39						
Node metastasis							
Positive	27	0.000	1.677 (0.370%)		0.004	2.892	1.326-5.278
Negative	35						

**Figure 2:**
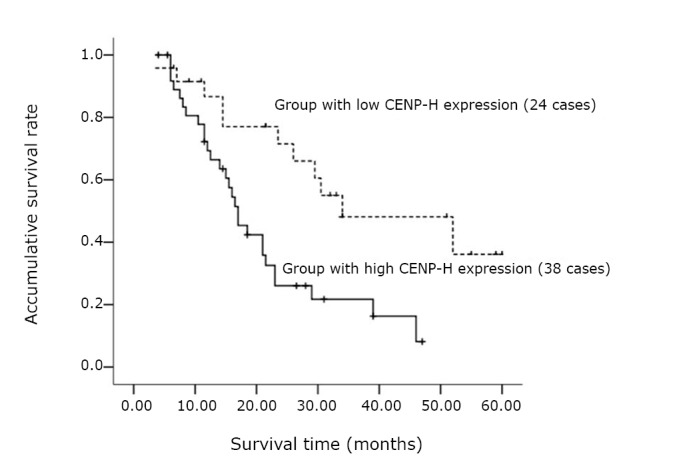
Comparison of the survival rates between the group with high CENP-H expression and that with low CENP-H expression in the 62 UCC patients.

Kaplan-Meier analysis revealed that there were significant differences in the survival time between the patients in the group with high CENP-H expression and those with low CENP-H expression (*P*=0.002). The median survival was 17 months for the patients with high CENP-H expression and 34 months for those with low CENP-H expression (**Figure 2**).

## Discussion

Uterine cervical cancer (UCC) is one of the most commonly seen tumor in gynecological oncology, and is the first cause of death in cancer patients of developing countries. In the past few years, there is a trend that UCC patients are younger and UCC incidence increases in patients younger than 35^[^^1^^]^. The onset and progression of cancer is a course in which multiple steps and genes are involved. The incidence and progression of UCC have experienced atypical hyperplasia at first, and then cancer in situ and infiltrating carcinoma. Therefore, research on the mechanism for onset and progression of UCC will be of great importance to the clinical diagnosis and treatment of this disease. Centromere is the structural and functional elements that assure normal cell division and separation of the chromosome to the daughter cells during the cellular mitosis and meiosis. At present it was known that there are more than 40 centromere proteins (CENP) in human body, and CENP-H is an important one among the CENP proteins. In the cell cycles, CENP-H, together with CENP-A and CENP-C, is located at the centromeric inner plate in the chromosome. The physiological function of the protein is to form the CENP-H-I complex by combining the centromeric inner plate protein such as CENP-I. Then, other CENPs such as CENP-F, Nuf2, MCAK and mitotic checkpoint proteins, such as MAD1, MAD2, BUB1, BUBR1, BUB3 and MPS1 were recruited for perfect combination with the centromere^[^^8^^]^, thus regulate the mitosis of the cells. The high expression or abnormal location of CENP-H may result in a chaotic mechanism of cell division, which brings about a phenomenon of the chromosomal aneuploidy. Chromosomal abnormalities, which includes chromosomal aneuploidy, is a hallmark of cancer^[^^9^^]^. The chromosomal aneuploidy is common in the tumor cells of human body and in the precancerosis of the tumors such as carcinoma of the esophagus. If the chromosomal aneuploidy occurs, it usually indicates cancerization.

In the past few years, high expression of CENP-H was found in colon cancer^[^^4^^]^, NPC^[^^6^^]^ and squamous cell carcinoma of the tongue^[^^5^^]^. It was shown in our study that compared with no expression or low expression of CENP-H in the mucosal tissues around the tumor, the expression rate of CENP-H was up to 61.29% in UCC. However, the high expression of CENP-H results in occurrence of the chromosome aneuploid in UCC cells. This study found that CENP-H expression and the scope of the primary focus of UCC was in close correlation with clinical staging, but was not associated with tumor metastasis. The greater the primary focus of UCC is, the stronger the CENP-H expression. CENP-H expression increased with the increase of clinical staging, suggesting that the high expression of CENP-H may play a role in the tumorigenesis and progression. There were no statistical differences in CENP-H expression between the groups with and without metastasis, indicating that CENP-H might not promote the tumor metastasis. This study found that the prognosis was very poor in patients with high CENP-H expression, with a median survival of 17 months. And the median survival was 34 months in patients with low CENP-H expression. It is thus concluded that there is an inverse relationship between the level of CENP-H expression and survival time of UCC patients. These results are in accordance with the conclusions in NPC^[^^6^^]^ and squamous cell carcinoma of the tongue^[^^5^^]^. Therefore, CENP-H can be considered as an index for assessing the prognosis of UCC patients.

In conclusion, there is a positive correlation between CENP-H and clinical staging of UCC, and a negative correlation between high CENP-H expression and prognosis of the patients. The detection of CENP-H expression is of importance in determining the biological behavior and prognosis of UCC, and is expected to be a novel target in the molecular targeted therapy of UCC.
